# The Defect in Autophagy Induction by Clinical Isolates of *Mycobacterium Tuberculosis* Is Correlated with Poor Tuberculosis Outcomes

**DOI:** 10.1371/journal.pone.0147810

**Published:** 2016-01-27

**Authors:** Furong Li, Bo Gao, Wei Xu, Ling Chen, Sidong Xiong

**Affiliations:** 1 Institute for Immunobiology, Department of Immunology, Shanghai Medical College of Fudan University, Shanghai 200032, P.R. China; 2 Jiangsu Key Laboratory of Infection and Immunity, Institutes of Biology and Medical Sciences, Soochow University, Suzhou 215006, P.R. China; 3 Department of Respiratory Medicine, Affiliated Hospital of Zunyi Medical College, Zunyi 563000, P.R. China; The Catholic University of the Sacred Heart, Rome, ITALY

## Abstract

**Background:**

Tuberculosis (TB) represents a major global health problem. The prognosis of clinically active tuberculosis depends on the complex interactions between *Mycobacterium tuberculosis* (*Mtb)* and its host. In recent years, autophagy receives particular attention for its role in host defense against intracellular pathogens, including *Mtb*. In present study, we aim to investigate the relationship of autophagy induction by clinical isolates of *Mtb* with the clinical outcomes in patients with TB.

**Methodology/Principal Findings:**

We collected 185 clinical isolates of *Mtb*, and determined the effect of these *Mtb* isolates on autophagy induction in macrophages. It was found that most of clinical isolates of *Mtb* were able to induce autophagosome formation in macrophages, however, the autophagy-inducing ability varied significantly among different isolates. Of importance, our results revealed that patients infected by *Mtb* with poor autophagy-inducing ability displayed more severe radiographic extent of disease (*p*<0.001), and were more likely to have unfavorable treatment outcomes (*p*<0.001). No significant association was observed between the extent of *Mtb*-induced autophagy with some socio-demographic characteristics (such as gender, age and tobacco consumption), and some laboratory tests (such as hemoglobin, leukocyte count and erythrocyte sedimentation rate). Furthermore, results from logistic regression analysis demonstrated that the defect in autophagy induction by clinical isolates of *Mtb* was an independent risk factor for far-advanced radiographic disease (aOR 4.710 [1.93–11.50]) and unfavorable treatment outcomes (aOR 8.309 [2.22–28.97]) in TB.

**Conclusion/Significance:**

These data indicated that the defect in autophagy induction by *Mtb* isolates increased the risk of poor clinical outcomes in TB patients, and detection of clinical isolates-induced autophagosome formation might help evaluate the TB outcomes.

## Introduction

Tuberculosis (TB), caused by the bacillus *Mycobacterium tuberculosis* (*Mtb*), is a major public health problem worldwide with up to 10 million new cases each year, leading to 1.5 million deaths annually. China occupies second place, behind India, among the top five high-burden countries for the last decade [1, 2]. The application of BCG vaccine and anti-TB antibiotics has been effective in preventing and controlling TB. However, the high rates of latent tuberculosis infection (LTBI), emergence of drug-resistant *Mtb* and HIV co-infection, etc., have made the control and treatment of TB become difficult in recent years [3–5]. Novel and effective therapeutic strategies against TB are therefore urgently needed. Accumulating evidence indicates that the interaction between *Mtb* and the host is of great importance in determining the outcome of TB [6–8]. Of those strategies targeting *Mtb*-host interaction, autophagy receives particular attention in recent years [9–12].

Autophagy is an evolutionarily conserved process in which organelles and proteins are sequestered into a double-membrane-bound autophagosome, and delivered to the lysosome for degradation. Recent reports reveal that autophagy is involved in diverse pathophysiological processes, including cell survival, aging, neurodegeneration, cancer and the clearance of intracellular pathogen [13–15]. Accumulating evidence indicates that autophagy may function as a crucial anti-TB strategy of the host, although these data are mainly obtained from the investigation on BCG or standard H37Rv strain. On the other hand, it is also suggested that through the intimate and persistent interaction with its human host, *Mtb* may have evolved strategies to counter the antibacterial effect of autophagy [9, 10, 12, 16–18]. The pathogenesis of TB has been thought to be mainly related to host factors in earlier investigations, however, it appears clear now that bacterial factors also play crucial roles [7]. Reports indicate that clinical isolates of *Mtb* display different characteristics from those of BCG or H37Rv [19, 20]. Different clinical isolates of *Mtb* are also found to induce different host immune responses [21, 22]. It is therefore of interest to further investigate the role of autophagy in TB pathogenesis using clinical isolates of *Mtb*.

In this study, we collected 185 clinical isolates of *Mtb* from Zunyi, one of the highest-incidence-rate areas with TB in China [23], and investigated the effect of these clinical isolates on autophagosome formation in macrophages, and its associated clinical significance. Our data showed that most clinical isolates of *Mtb* were able to induce autophagosome formation in macrophages, however different clinical isolates of *Mtb* differed in their ability to induce autophagosome formation. Of importance, it was found that the extent of clinical isolates of *Mtb*-induced autophagy was negatively correlated with the clinical outcomes in patients with TB.

## Materials and Methods

### Mycobacterial specimens

A total of 185 *Mtb* isolates were obtained from clinical patients, from 2011 to 2014 in Zunyi, Guizhou Province, one of the highest-incidence-rate areas with TB in China. Of these samples, 177 specimens were from sputum, 6 from urine, 1 from the cerebrospinal fluid, and 1 from the scrotum. *Mtb* isolates were grown in 7H11 medium (Difco BD, NY) supplemented with 0.05% Tween 80 and 10% oleic albumin dextrose catalase enrichment (Difco, Detroit, MI), and the identification of all these isolates were performed according to the TB diagnosis bacteriology test criteria of the China Antituberculosis Association. Single-cell suspensions of mycobacteria at a concentration of 10^7^ CFU/ml were prepared and used to infect cells.

### Data collection

Following verbal and written consent, socio-demographic, clinical, radiographic and laboratory data were obtained from patients' medical record, and the data were analyzed anonymously. The Ethics Committee of Fudan University and Affiliated Hospital of Zunyi Medical College specifically approved this study, and this work was also performed in compliance with the Helsinki Declaration. The radiographic extent of disease was categorized to be "minimal", "moderately advanced", or "far advanced" according to the classification of the National Tuberculosis and Respiratory Disease Association [24]. The TB treatment outcomes were defined by WHO criteria as "favourable" (cured and treatment completed) and "unfavourable" (defaulted, failed and died) [25]. Retreatment cases were those having history of previous TB treatment of more than one month.

### Macrophage stimulation with mycobacterial strains

The murine macrophage cell line RAW264.7 (ATCC number: TIB-71) was maintained at 37°C in DMEM (Invitrogen, Carlsbad, CA, USA) supplemented with 10% FBS (HyClone, Logan, UT, USA) and antibiotics in a 5% CO_2_ atmosphere. The human monocyte cell line THP-1 (ATCC number: TIB-202) was cultured in RPMI1640 (Invitrogen) supplemented with 10% FBS (HyClone). Prior to *Mtb* infection, THP-1 cells were treated with 50 ng/ml Phorbol 12-myristate 13-acetate (PMA) for 24 hours to allow differentiation into macrophages. BMDM of > 95% purity were obtained from BALB/c as described previously [26]. Macrophage stimulation with *Mtb* strains was performed according to previous reports [16, 27]. Briefly, macrophages were infected with clinical isolates of *Mtb* or H37Rv at multiplicity of infection (MOI) of 10:1. Four hours after infection, macrophages were washed twice with prewarmed serum-free RPMI1640 or DMEM to remove unbound bacilli, and were further cultured in serum-supplemented RPMI1640 or DMEM for another 4 hours. No toxicity was observed in *Mtb*-infected macrophages.

### Western blot analysis

Western blot was performed as described previously [28]. Antibody against LC3 was obtained from Sigma (St. Louis, Mo, USA), and anti-GAPDH was from CWBio (Beijing, China).

### Immunofluorescence

THP-1 cells were washed twice with PBS, fixed with 4% paraformaldehyde in PBS for 10 min, permeabilized with 0.25% Triton X-100 in PBS for 10 min. Cells were then incubated sequentially with rabbit anti-LC3 antibody and tetramethyl rhodamine isothiocyanate (TRITC)-conjugated goat anti-rabbit IgG (red), followed by the staining with 4',6-diamidino-2-phenylindole (DAPI) to visualize the nuclei (blue). Fluorescence images were acquired with a confocal laser-scanning microscope.

### Statistical analysis

Categorical variables were compared using pearson chi-square test, and the difference in continuous variables was analyzed by one-way analysis of variance (One-way ANOVA). Variables with a *p*-value < 0.2 in univariate logistic regression analyses were further subjected to multivariate logistic regression analysis to identify independent variables that evaluated the role of autophagy in the pathogenesis of TB. A *p*-value <0.05 was considered to be statistically significant. Data were entered and analyzed using a statistical software package (SPSS18.0).

## Results

### Clinical isolates of *Mtb* were able to induce autophagosome formation in macrophages

**Fig 1:**
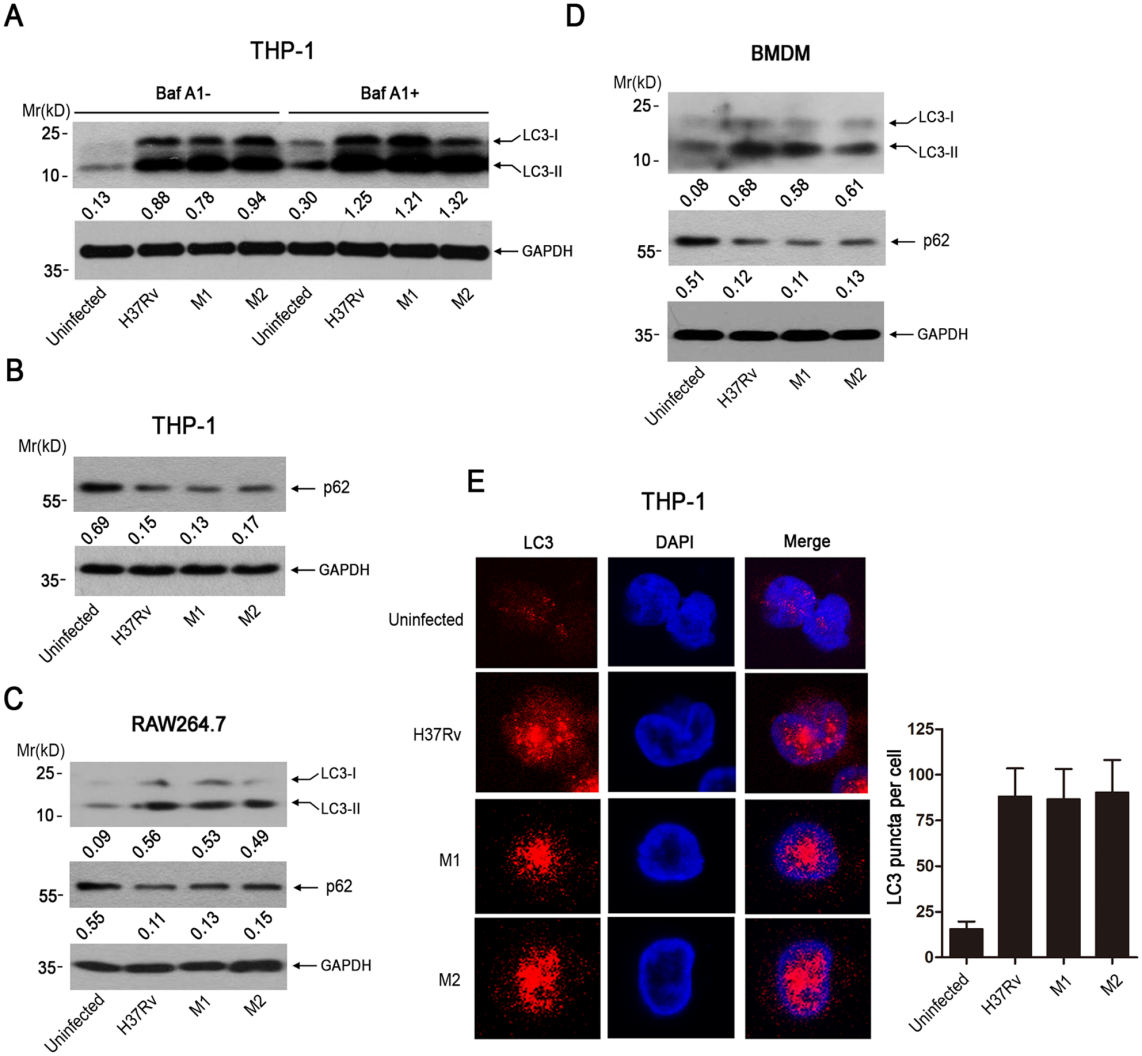
Clinical isolates of *Mtb* could induce autophagy in macrophages. (A) THP-1 were infected with H37Rv and two clinical isolates of *Mtb* (M1 and M2) (MOI = 10) for 4 hr in the absence or presence of Baf A1. Cells were harvested and subjected to western blot analysis using anti-LC3. The expression of GAPDH was used as a loading control. The immunoblots were scanned and subjected to densitometric analysis. LC3-II/GAPDH ratio was calculated, and the mean value of at least five samples from three independent experiments was shown at the bottom of each lane. (B) THP-1 were infected with clinical *Mtb* isolates (M1, M2) or H37Rv (MOI = 10) for 4 hr. Cells were harvested and subjected to western blot analysis using antibodies against p62 and GAPDH. Protein was quantitified by densitometry. P62/GAPDH ratio was calculated, and the mean value of at least five samples from three independent experiments was shown at the bottom of each lane. (C and D) RAW264.7 and BMDMs macrophages were treated as in B. Cells were harvested and subjected to western blot analysis using antibodies against p62 and GAPDH. Protein was quantitified by densitometry. LC3-II/GAPDH or p62/GAPDH ratio was calculated, respectively, and the mean value of at least five samples from three independent experiments was shown at the bottom of each lane. (E) THP-1 macrophages were infected with a clinical isolates of *Mtb* (MOI = 10) for 4 h. Cells were then incubated sequentially with anti-LC3B antibody and TRITC goat anti-rabbit IgG (red), followed by the staining with DAPI to visualize the nuclei (blue), the right panel was the quantification of LC3 punctuate per cell. The data shown represent mean ± SE from three independent experiments.

To investigate the effect of *Mtb* on autophagy induction, we infected human THP-1 macrophages with two clinical isolates of *Mtb* or H37Rv for 4 hr, followed by detecting the autophagosome formation using western blot. Results showed that, similar to H37Rv, two clinical isolates of *Mtb* (M1, M2) were able to significantly increase the level of LC3 (microtubule-associated protein light chain 3)-II, a hallmark of autophagy induction (Fig 1A). As the the increase in LC3II level could mean that there is an increase in autophagy induction or there is an inhibition of flux, we examined the effect of Bafilomycin A1 (Baf A1) treatment on *Mtbs*-induced autophagy. It was found that Baf A1 treatment could further increase the LC3II level in *Mtbs*-infected THP1 cells (Fig 1A). We have also investigated the effect of *Mtbs* infection on the expression level of p62, which is incorporated into autophagosomes and degraded along with other substrates by lysosomal hydrolyses. Our results showed that infection with clinical isolates (M1, M2) or H37Rv led to the downregulation of p62 significantly in THP1 cells at 4 hours postinfection (Fig 1B). Together, these data indicated that these *Mtbs*-induced autophagy was functional rather than blocking autophagical flux at 4 hours postinfection. We also examined the effect of clinical isolates of *Mtb* on autophagy induction in mouse macrophage RAW264.7 cells and mouse bone marrow-derived macrophages (BMDM). Similar to the observation in THP-1 cells, infection with clinical isolates (M1, M2) or H37Rv led to the increase in LC3II level while downregulated p62 expression in RAW264.7 cells (Fig 1C) and BMDM cells (Fig 1D). We further determined the effect of clinical *Mtb* isolate on autophagosome formation using the immunofluorescence technique. The confocal result showed that clinical *Mtb* isolates (M1, M2) or H37Rv stimulated the formation of LC3 punctuate in THP-1 cells significantly (Fig 1E).

### Different clinical isolates of *Mtb* induced autophagosome formation in macrophages to different extent

**Fig 2:**
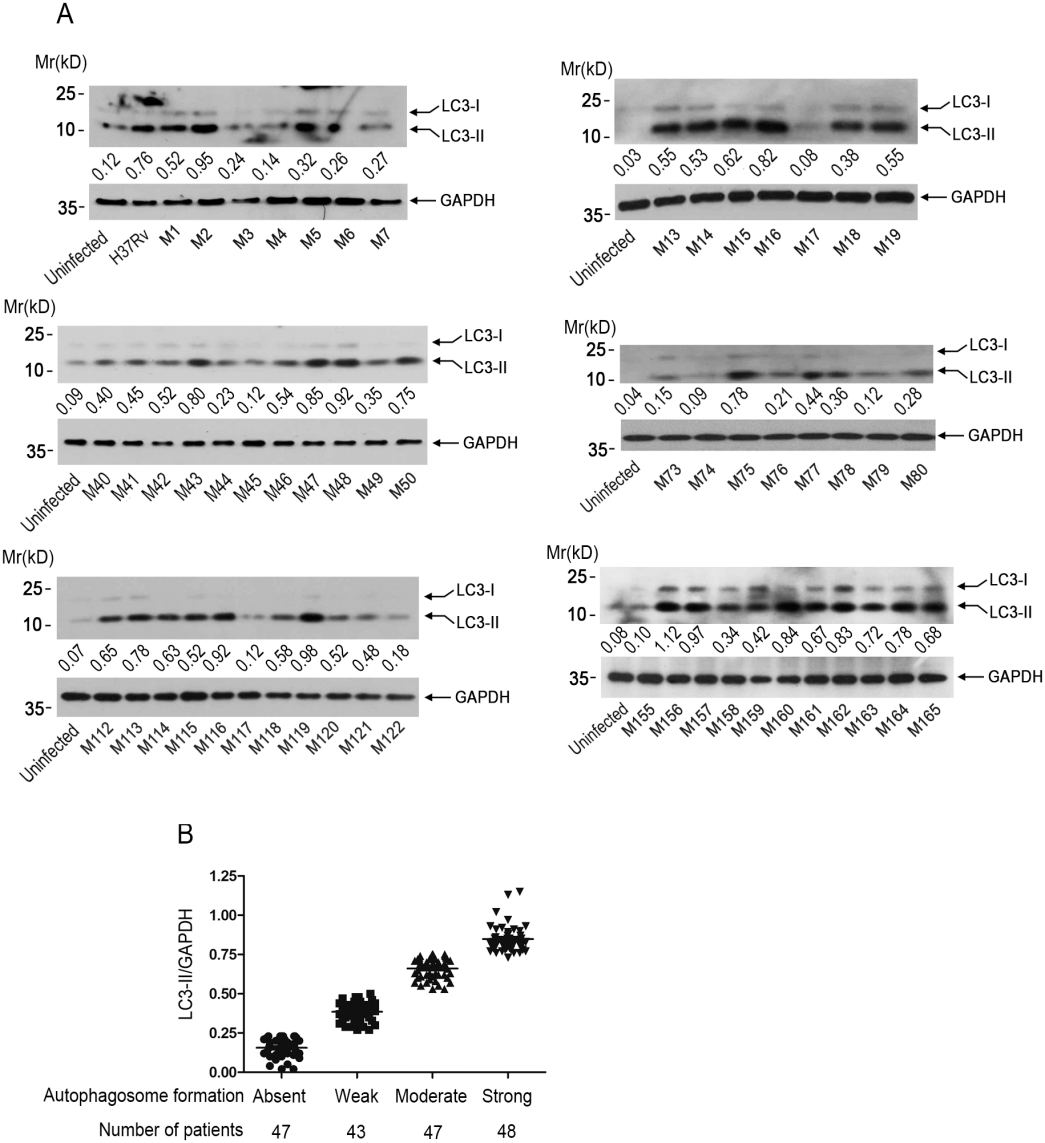
Different clinical isolates of *Mtb* induced autophagy in THP-1 cells to a different extent. **(A)** THP-1 cells were infected with clinical isolates of *Mtb* for 4 hr. Cells were harvested and subjected to western blot analysis using anti-LC3. GAPDH was used as a loading control. Representative immunoblots (sample 1–7, 13–19, 40–50, 73–80, 112–122, 155–165) were shown. The immunoblots were scanned and subjected to densitometric analysis. LC3-II/GAPDH ratio was calculated, and the mean value of at least five samples from three independent experiments was shown at the bottom of each lane. **(B)** The extent of autophagosome formation was graded on LC3-II/GAPDH ratio into four classes: absent (0–0.25), weak (0.26–0.50), moderate (0.51–0.75), and strong (>0.75).

Above data showed that two clinical isolates of *Mtb* were able to induce autophagosome formation in macrophages, we therefore further collected more clinical isolates of *Mtb* to a total of 185 during the period from 2011 to 2014, and examined the effect of these clinical isolates of *Mtb* on autophagosome formation in THP-1 macrophages using western blot. It was found that most of these clinical isolates of *Mtb* could induce autophagosome formation, however, the autophagy-inducing ability appeared to vary greatly among different isolates (Fig 2A). To better define the clinical isolates of *Mtb*-induced autophagosome formation, the extent of autophagosome formation was graded on LC3-II/GAPDH ratio into four classes: absent (0–0.25), weak (0.26–0.50), moderate (0.51–0.75), and strong (>0.75) (Fig 2B).

### Characteristics of patients harbouring clinical isolates of *Mtb* with different autophagy-inducing ability

As above data demonstrated that clinical isolates of *Mtb* differed in their ability to induce autophagosome formation in THP-1 macrophages and autophagy were reported to be crucial for *Mtb* clearance, we would like to know the relationship between the extent of *Mtb*-induced autophagosome formation and the clinical outcomes of TB patients. The clinicopathological comparisons among patients harbouring clinical isolates of *Mtb* with different autophagy-inducing ability were shown in Table 1. It was found that patients infected by *Mtb* with poor autophagy-inducing ability displayed more severe radiographic extent of disease (*p*<0.001) and were more likely to have unfavorable treatment outcomes (*p*<0.001). We also observed that re-treatment TB cases were more likely to harbour isolates with poor autophagy-inducing ability (*p*<0.001). There was no significant association of the extent of *Mtb*-induced autophagy with some socio-demographic characteristics (including gender, age, tobacco smoking and alcohol consumption), the coexistence of pulmonary and extra pulmonary tuberculosis (PTB+EPTB), and some laboratory tests [such as hemoglobin, leukocyte count, erythrocyte sedimentation rate (ESR), and carbon dioxide combining power (CO_2_CP)].

**Table 1:** Characteristics of patients harboring clinical Mtb isolates with different autophagy-inducing ability.

Variable		Autophagosome formation				
	Total	Absent	Weak	Moderate	Strong	*P-*value^a^
	N = 185 n (%)	N = 47 n (%)	N = 43 n (%)	N = 47 n (%)	N = 48 n (%)	
**Gender**						**0.098**
Male	106 (57.3)	21 (44.7)	25 (58.1)	33 (70.0)	27 (57.3)	
Female	79 (42.7)	26 (55.3)	18 (61.9)	14 (30.0)	21 (42.7)	
**Age (years)**						**0.334**
<30	40 (21.6)	11 (23.4)	14 (32.6)	8 (17.0)	7 (14.6)	
30–50	88 (47.6)	21 (44.7)	16 (37.2)	27 (57.4)	24 (50.0)	
>50	57 (30.8)	15 (31.9)	13 (30.2)	12 (25.6)	17 (35.4)	
**Tobacco**						**0.161**
Yes	86 (46.5)	27 (57.4)	22 (51.2)	20 (42.6)	17 (35.4)	
No	99 (53.5)	20 (42.6)	21 (48.8)	27 (57.4)	31 (64.6)	
**Alcohol**						**0.561**
Yes	91 (49.2)	25 (53.2)	18 (41.9)	24 (51.1)	27 (56.3)	
No	94 (50.8)	22 (46.8)	25 (58.1)	23 (48.9)	21 (43.7)	
**TB form**						**0.889**
PTB alone	131 (70.8)	35 (74.5)	28 (65.1)	32 (68.1)	36 (75.0)	
EPTB alone	8 (4.3)	1 (2.1)	2 (4.7)	3 (6.4)	2 (4.2)	
PTB+EPTB	46 (24.9)	11 (23.4)	13 (30.2)	12 (25.5)	10 (20.8)	
**Radiographic extent of disease**						**0.001**
Minimal	53 (28.6)	7 (14.9)	9 (20.9)	16 (34.0)	21 (43.8)	
Moderate-advanced	70 (37.8)	14 (29.8)	16 (37.2)	21 (44.7)	19 (39.6)	
Far-advanced	62 (33.6)	26 (55.3)	18 (41.9)	10 (21.3)	8 (16.6)	
**Laboratory testings**						
**Hemoglobin (g/L)**	129.4±18.3	128.2±16.3	132.6±18.4	126.8±17.9	130.3±20.5	**0.463**^b^
**Leukocytes (10**^**9**^**/L)**	7.8±2.7	7.1±2.4	8.15±2.5	8.3±3.1	7.6±2.6	**0.607**^b^
**ESR (mm)**	48.2±21.4	48.5±20.8	49.0±20.4	49.3±21.4	45.9±23.1	**0.865**^b^
**CO**_**2**_**CP (mmol/L)**	24.9±3.5	25.2±3.3	24.8±3.7	24.6±3.5	25.3±3.7	**0.720**^b^
**Type of cases**						**<0.001**
New	99 (53.5)	12 (25.5)	17(39.5)	31 (66.0)	37 (77.1	
Re-treatment	86 (46.5)	35 (74.5)	26(60.5)	16 (34.0)	11 (22.9)	
**Treatment outcomes**						**<0.001**
Favourable *(cured*, *completed)*	145 (78.4)	29 (61.7)	30 (69.8)	40 (85.1)	46 (95.8)	
Unfavourable *(failed*,*defaulted*,*died)*	40 (21.6)	18 (38.3)	13 (30.2)	7 (14.9)	2 (4.2)	

Abbreviation: PTB, pulmonary tuberculosis; EPTB, extra-pulmonary tuberculosis; ESR, erythrocyte sedimentation rate; CO2CP, Carbon Dioxide Combining Power

^a^Chi-square test

^b^One way ANOVA.

### The defect in autophagy induction by *Mtb* increased the risk of far-advanced radiographic disease and unfavorable treatment outcomes in TB patients

Results from Table 1 showed that there was a higher portion of patients with far-advanced radiographic disease in the poor autophagy-inducing group as compared with the strong autophagy-inducing group. We further determined the association between them by logistic regression analysis. The univariate analysis showed that the defect in *Mtb* isolates-induced autophagosome formation was positively correlated with the far-advanced radiographic disease [odd ration (OR), 4.574; 95% confidence interval (CI) 1.93–10.86; p = 0.001]. When controlling for age, tobacco consumption and ESR, the poor autophagy-inducing ability by *Mtb* remained as a significant risk factor for the far-advanced radiographic disease [adjusted OR (aOR), 4.710; 95% CI 1.93–11.50; p = 0.001]. Besides, over 50 years of age was also revealed to be associated with the far-advanced radiographic disease (aOR, 2.915; 95% CI 1.12–7.58; *p* = 0.028) in the final regression model. Details are showed in Table 2.

**Table 2:** Univariate and multivariate logistic regression analysis of far-advanced radiographic disease in TB patients*.

Variable	Radiographic finding				
		Far-advanced*		Univariate analysis	Multivariate Analysis
	All	n	%	OR (95%CI), *P*-value	aOR (95%CI), *P*-value
**Autophagosome formation**					
Strong	48	8	16.7	Reference	Reference
Moderate	47	10	21.3	1.351 (0.48–3.79), 0.567	1.303 (0.46–3.72), 0.621
Absent-Low	90	43	47.8	4.574 (1.93–10.86), 0.001	4.710 (1.93–11.50), 0.001
**Gender**					
Female	79	25	31.7	Reference	
Male	106	36	34	1.005 (0.54–1.87), 0.988	
**Age**					
<30	40	10	25	Reference	Reference
30–50	88	28	31.8	1.400 (0.60–3.26), 0.435	2.089 (0.85–5.16), 0.110
>50	57	23	40.4	2.029 (0.83–4.94), 0.119	2.915(1.12–7.58), 0.028
**Alcohol**					
No	94	33	35.1	Reference	
Yes	91	28	30.8	0.822 (0.44–1.52), 0.531	
**Tobacco**					
No	99	28	28.3	Reference	Reference
Yes	86	33	38.4	1.579 (0.85–2.93), 0.147	1.381 (0.71–2.69), 0.344
**TB form**					
PTB alone	131	45	34.4	Reference	
PTB+EPTB	46	16	34.8	1.019 (0.50–2.07), 0.958	
**Leukocytes (10**^**9**^**/L)**					
≤10.0	152	50	32.9	Reference	
>10.0	33	11	33.3	0.980 (0.44–2.18), 0.961	
**ESR (mm)**					
≤40	77	21	27.3	Reference	Reference
>40	108	40	37	1.569 (0.83–2.96), 0.165	1.609 (0.81–3.19), 0.174

Abbreviation: OR, odd ratio; aOR, adjusted OR; CI, confidence interval

*For the regression analysis of radiographic findings, the radiographic extent of disease was categorized into two group: Minimal to moderate-advanced group and Far-advanced group.

We further investigated the correlation between the extents of *Mtb*-induced autophagosome formation with the treatment outcome of TB patient. Result from the univariate analysis showed that the defect in *Mtb*-induced autophagosome formation was positively correlated with the unfavorable treatment outcomes of TB patients (OR, 7.881; 95% CI 2.27–27.43; *p* = 0.001). In further multivariate analysis adjusted for age, tobacco smoking and leucocyte count, the poor autophagy-inducing ability by *Mtb* remained as a significant risk factor for unfavorable outcomes in TB patients (aOR, 8.310; 95% CI 2.22–28.97; *p* = 0.001). Of the covariates included in the final model, over 50 years of age (aOR, 4.274; 95% CI 1.32–13.86; *p* = 0.015) and PTB+EPTB (aOR, 2.504; 95% CI 1.18–5.33; *p* = 0.031) were also revealed as the risk factors for unfavorable treatment outcomes in TB patients. Details are presented in Table 3.

**Table 3:** Univariate and multivariate logistic regression analysis of treatment outcomes in TB patients.

Variable	Treatment outcome				
		Unfavorable		Univariate analysis	Multivariate Analysis
	All	n	%	OR (95%CI), *P*-value	aOR (95%CI), *P*-value
**Autophagosome formation**					
Strong	48	3	6.3	Reference	Reference
Moderate	47	7	14.9	2.625 (0.64–10.84), 0.182	2.412 (0.57–10.30), 0.235
Absent-Low	90	31	34.4	7.881 (2.27–27.43), 0.001	8.024 (2.22–28.97), 0.001
**Gender**					
Female	79	15	19	Reference	
Male	106	26	24.5	1.387 (0.68–2.84), 0.371	
**Age**					
<30	40	5	12.5	Reference	Reference
30–50	88	19	21.6	1.928 (0.66–5.60), 0.228	2.730 (0.88–8.48), 0.083
>50	57	14	24.6	2.975 (0.99–8.90), 0.051	4.274 (1.32–13.86), 0.015
**Alcohol**					
No	94	19	20.2	Reference	
Yes	91	22	24.2	1.259 (0.63–2.52), 0.517	
**Tobacco**					
No	99	18	18.2	Reference	Reference
Yes	86	23	26.7	1.643 (0.82–3.31), 0.164	1.616 (0.73–3.57), 0.318
**TB form**					
PTB alone	131	22	16.8	Reference	Reference
EPTB alone	8	2	25	1.585 (0.30–8.25), 0.597	2.003 (0.29–12.85), 0.494
PTB+EPTB	46	16	34.8	2.504 (1.18–5.33), 0.017	2.525 (1.09–5.85), 0.031
**Leukocytes (10**^**9**^**/L)**					
≤10.0	152	32	21.1	Reference	
>10.0	33	9	27.3	1.406 (0.60–3.32), 0.437	
**ESR (mm)**					
≤40	77	12	15.6	Reference	Reference
>40	108	29	26.9	1.99 (0.94–4.20), 0.072	2.104 (0.92–4.80), 0.077

Abbreviation: PTB, pulmonary tuberculosis; EPTB, extra-pulmonary tuberculosis; OR, odd ratio; aOR, adjusted OR; CI, confidence interval.

Together, these data indicated that the defect in autophagy induction by *Mtb* posed as an independent risk factor for poor clinical outcomes in patients with TB.

## Discussion

The current study revealed that most of clinical isolates of *Mtb* was able to induce autophagosome formation in macrophages. The results also revealed that clinical isolates of *Mtb* differed significantly in their ability to induce autophagosome formation. Furthermore, our data revealed that the defect in autophagy induction by clinical isolates was positively correlated with the poor clinical outcomes in TB patients.

Autophagy is revealed to play a crucial role in host defense against *Mtb* by both *in vitro* and *in vivo* investigation [9, 12, 16]. Results from a genome-wide analysis of the host intracellular network also indicate that autophagy is implicated in the regulation of *Mtb* survival [29]. Autophagy may contribute to the elimination of *Mtb* through the fusion of *Mtb*-containing autophagosomes with lysosomes, leading to the xenophagic degradation of *Mtb*, and/or the enhancement of antigen presentation, and the consequent activation of adaptive immunity. On the other hand, through long battles with the host, *Mtb* may have developed various strategies to evade the autophagy-mediated antibacterial activities [11, 14]. In present investigation, we focus on the effect of clinical isolates of *Mtb* on autophagy induction and its possible association with clinical outcomes in TB patients.

We found that although most of clinical isolates of *Mtb* could induce autophagy in macrophages, the autophagy-inducing ability varied significantly among these clinical isolates. Reports indicate that the genotype of *Mtb* may have an important role in its behaviors [23, 30, 31], we thus would like to know whether the autophagy-inducing ability of these clinical isolates was related to their genetic background. We examined the genotypes of these *Mtb* isolates by the mycobacterial interspersed repetitive unit-variable number of tandem repeats (MIRU-VNTR) technique, and determined the possible association between the genotype and autophagy induction by cluster analysis using BioNumerics software. Our results revealed that there was no significant correlation between the clinical isolates-induced autophagosome formation and their genotypes (data not shown). We further investigated the association between the extent of autophagy induction by clinical isolates of *Mtb* with various clinical variables, including the socio-demographic characters, radiographic findings, laboratory tests and treatment outcomes, etc. Our results showed that that the defect in autophagy induction by clinical isolates was an independent risk factor for far-advanced radiographic disease and unfavorable treatment outcomes in TB patients. Additionally, our data also identified the *Mtb*-induced autophagosome formation was an independent risk factor for re-treatment TB cases (OR, 8.754; 95% CI 3.64–21.08; *p*<0.001) (S1 Table). Collectively, these data indicated that the autophagy-inducing ability by clinical isolates of *Mtb* might play a crucial role in determining the clinical outcomes in TB patients.

Given the importance of clinical isolates of *Mtb*-induced autophagy in TB outcomes, an interesting question was raised. Why different clinical isolates of *Mtb* possessed different ability in inducing autophagosome formation. This work was now undertaken in our laboratory. Reports indicated that H37Rv infection could suppress the autophagy flux in macrophages [17, 18], however, which could be rescued by *Mtb*-specific T cells [17]. We think that, besides the manipulation of autophagy induction, clinical isolates of *Mtb* may have developed other strategies for their survival. It is of interest in further investigation to determine whether clinical isolates of *Mtb* could impair autophagy flux, and the possible involvement of host factors, such as *Mtb*-specific T cells, in this process.

In conclusion, this study revealed that clinical isolates of *Mtb* differed in their ability to induce autophagy, which was closely correlated with clinical outcomes in TB patients, indicating the control of autophagy induction might be an important strategy manipulated by the bacteria to evade host immune responses. To our knowledge, this is the first report investigating the association between the autophagy induction and TB outcomes using clinical isolates. These data may help better understand the role of autophagy in the pathogenesis of tuberculosis, and provide important information for the better control of TB infection.

## Supporting Information

S1 Table Univariate and multivariate logistic regression analysis of retreatment TB cases. (DOC)

S2 Table Raw data of patients’ characteristics. (XLSX)
