# Supplementary material for: The Defect in Autophagy Induction by Clinical Isolates of Mycobacterium Tuberculosis Is Correlated with Poor Tuberculosis Outcomes
Source: PLoS One. 2016 Jan 27;11(1):e0147810. doi: 10.1371/journal.pone.0147810 (PMC4729487; doi:10.1371/journal.pone.0147810)
Supplement: S1 Table — (DOC) [file pone.0147810.s001.doc]

| **S1 Table. Univariate and multivariate logistic regression analysis of retreatment TB cases** | | | | | |
| --- | --- | --- | --- | --- | --- |
| **Variable** | **Type of cases** | | | **Univariate analysis** | **Multivariate analysis** |
|  | All | Retreatment | |  |  |
|  |  | n | % | OR (95%CI), *P*-value | aOR(95%CI), *P*-value |
| **Autophagosome formation** | | | | | |
| Strong | 48 | 11 | 22.9 | Reference | Reference |
| Moderate | 47 | 16 | 34.0 | 1.736 (0.70-4.29), 0.232 | 1.547 (0.60-4.00), 0.368 |
| Absent-Low | 90 | 60 | 66.7 | 7.075 (3.16-15.83), ＜0.001 | 8.937 (3.73-21.44), ＜0.001 |
| **Gender** | | | | | |
| Female | 79 | 37 | 46.8 | Reference |  |
| Male | 106 | 51 | 48.1 | 1.053 (0.587-1.89), 0.863 |  |
| **Age** | | | | | |
| ＜30 | 40 | 15 | 37.5 | Reference | Reference |
| 30-50 | 88 | 42 | 47.7 | 1.522 (0.71-3.27), 0.282 | 2.717(1.12-6.62), 0.028 |
| ＞50 | 57 | 31 | 54.4 | 1.987 (0.87-4.54), 0.103 | 3.380 (1.30-8.80), 0.013 |
| **Alcohol** | | | | | |
| No | 94 | 43 | 45.7 | Reference |  |
| Yes | 91 | 45 | 49.5 | 1.160 (0.65-2.07), 0.614 |  |
| **Tobacco** | | | | | |
| No | 99 | 42 | 42.4 | Reference | Reference |
| Yes | 86 | 46 | 53.5 | 1.561 (0.87-2.79), 0.134 | 1.234 (0.63-2.42), 0.539 |
| **TB form** | | | | | |
| PTB alone | 131 | 59 | 45.0 | Reference |  |
| EPTB alone | 8 | 3 | 37.5 | 0.732 (0.17-3.19), 0.678 |  |
| PTB+EPTB | 46 | 26 | 56.5 | 1.586 (0.81-3.12), 0.182 |  |
| **Leukocytes (10^9^/L)** | | | | | |
| ≤10.0 | 152 | 68 | 44.7 | Reference | Reference |
| ＞10.0 | 33 | 20 | 60.6 | 1.900 (0.88-4.10), 0.101 | 2.688 (1.12-6.44), 0.027 |
| **ESR (mm)** | | | | | |
| ≤40 | 77 | 32 | 41.6 | Reference |  |
| ＞40 | 108 | 56 | 51.8 | 1.514 (0.84-2.73), 0.168 | 1.548(0.79-3.04), 0.205 |
| PTB, pulmonary tuberculosis; EPTB, extra-pulmonary tuberculosis; OR, odd ratio; aOR, adjusted OR; CI, confidence interval | | | | | |
